# INTEREST GROUP SESSION—REMINISCENCE, LIFE STORY AND NARRATIVE: RESEARCH AND PRACTICE: WISDOM OF THE ELDERS: ART, MEDICINE, NARRATIVES, AND POETRY

**DOI:** 10.1093/geroni/igz038.2102

**Published:** 2019-11-08

**Authors:** Ifeoma Nwankwo, Monika Ardelt

**Affiliations:** 1 Vanderbilt University, Nashville, Tennessee, United States; 2 University of Florida, Gainesville, Florida, United States

## Abstract

The Wisdom of the Elders (WOE) is a series of autobiography production workshops conducted since 2012 through a collaboration among community, university, and municipal partners. Beginning in Murfreesboro, TN it has since been replicated in New Orleans, LA. The Wisdom of the Elders project has many elements, including life history interviews, creative writing workshops, and creative expressions that allowed elders to share their wisdom with younger generations. Our focus was to understand how personal characteristics and historical/environmental events interact to influence healthy aging. Later work extended to interviewing older hospitalized patients in order to gain insight into intergenerational themes. The interdisciplinary team has also used poetry and reflection to train healthcare professionals and caregivers to use active listening strategies to better understand how patients cope with life-changing illnesses, and how they incorporate the challenges of those illnesses into rich, fulfilling lives. WOE has been supported by the Mellon Foundation, the Meharry-Vanderbilt Community Engaged Research Core, and the Department of English, the College of Arts and Science Dean’s Office, and the Chancellor’s Higher Education Fellowship at Vanderbilt University. WOE has led to the formation of an interdisciplinary research and publication collaborative featuring distinguished clinicians, artists, and scholars from Creative Writing, Education, Geriatrics, Interprofessional Learning, Psychology, and Public Humanities.

